# Structural basis for functional selectivity and ligand recognition revealed by crystal structures of human secreted phospholipase A_2_ group IIE

**DOI:** 10.1038/s41598-017-11219-8

**Published:** 2017-09-07

**Authors:** Shulin Hou, Tingting Xu, Jinxin Xu, Linbing Qu, Yong Xu, Ling Chen, Jinsong Liu

**Affiliations:** 1 State Key Laboratory of Respiratory Disease, Guangzhou Institutes of Biomedicine and Health, Chinese Academy of Sciences, Guangzhou, 510530 China; 2 University of Chinese Academy of Sciences, Beijing, 100000 China; 3 School of Life Sciences, University of Science and Technology of China, Hefei, 230026 China; 4 Guangdong Provincial Key Laboratory of Biocomputing, Institute of Chemical Biology, Guangzhou Institutes of Biomedicine and Health, Chinese Academy of Sciences, Guangzhou, 510530 China

## Abstract

Secreted phospholipases A_2_s (sPLA_2_s) are involved in various pathological conditions such as rheumatoid arthritis and cardiovascular disease. Many inhibitors were developed and studied in clinical trials, but none have reached the market yet. This failure may be attributed to the lack of subtype selectivity for these inhibitors. Therefore, more structural information for subtype sPLA_2_ is needed to guide the selective inhibitor development. In this study, the crystal structure of human sPLA_2_ Group IIE (hGIIE), coupled with mutagenesis experiments, proved that the flexible second calcium binding site and residue Asn21 in hGIIE are essential to its enzymatic activity. Five inhibitor bound hGIIE complex structures revealed the key residues (Asn21 and Gly6) of hGIIE that are responsible for interacting with inhibitors, and illustrated the difference in the inhibitor binding pocket with other sPLA_2_s. This will facilitate the structure-based design of sPLA_2_’s selective inhibitors.

## Introduction

The mammalian family of secreted phospholipase A_2_ (sPLA_2_), which are Ca^2+^ dependent, low-molecular weight and disulfide-rich enzymes, plays key roles in many physiological functions and pathological processes by catalyzing the hydrolysis of phospholipids at the sn-2 position^1, 2^. With the release of free fatty acid and lysophospholipid from cellular or non-cellular phospholipids, sPLA_2_s catalyzed reactions can lead to the production of various types of lipid signaling mediators, such as prostaglandins, leukotrienes and other eicosanoids^3, 4^. sPLA_2_ also can participate in the biological function by binding to the sPLA_2_ receptor and other proteins^5^.

The mammalian sPLA_2_ family consists of 11 members: GIB, GIIA, GIIC, GIID, GIIE, GIIF, GIII, GV, GX, GXIIA and GXIIB. They have distinct tissue and cellular distributions and substrate preference associated with their physiological functions^6^. GIB, which is abundantly expressed in the pancreas, is referred to as a “digestive sPLA_2_”. Gene disruption of GIB (*g1b* ^−/−^) in mice can protect against diet-induced obesity, hyperlipidemia and type 2 diabetes^7^. GIIA is often regarded as the “inflammatory sPLA_2_”, since its expression in serum or inflammatory exudates can be induced in the severity of inflammation^8^. GIIA is also regarded as the “bactericidal sPLA_2_”, owing to its high hydrolytic activity for phosphatidylglycerol (PG) and phosphatidylethanolamine (PE), which are abundant in bacterial membranes^9^. GIID is preferentially expressed in dendritic cells and macrophages, and displays a pro-resolution function. Therefore, GIID is regarded as the “resolving sPLA_2_” and can ameliorate inflammation through mobilizing pro-resolving/anti-inflammatory lipid mediators^10^. Among sPLA_2_s, GX has the highest activity for phosphatidylcholine (PC)^11^ and thus exhibits the most potent ability to hydrolyze the outer leaflet of the plasma membrane in intact cells^12^. GX can induce airway inflammation by acting as infiltrating eosinophils to augment leukotriene production in a process involving LysoPC-dependent activation of cytosolic PLA_2_α^13^.

GIIE, the closest homolog to GIIA, is regarded as a “hair follicular”, “inflammatory” and “metabolic” sPLA_2_. As a “hair follicular” sPLA_2_, GIIE in mouse is expressed abundantly in hair follicles, and mice lacking GIIE exhibit skin abnormalities^14^. GIIE has been reported to play a pivotal role in the inflammatory process. Expression of GIIE is highly upregulated in mice injected with lipopolysaccharide. GIIE enhances leukotriene production and granule exocytosis in mastocytoma cells^15^. GIIE is proved to be linked with metabolic disorders. Expression of GIIE is robustly induced in adipocytes of obese mice. High fat diet induced fatty liver is mildly ameliorated and the hepatic lipid deposition is also reduced in *g2e* ^−/−^ mice. *g2e* ^−/−^ mice contain more PE and PS (phosphatidylserine) species than *g2e* ^+/+^ mice, while PC species are similar in both genotypes. GIIE mildly promotes adiposity and fatty liver by altering the proportion of PE and PS in lipoproteins^16^. However there is some controversy about the metabolic function of GIIE. It is reported in a different study that triglyceride was accumulated in adipose tissue isolated from *g2e* ^−/−^ mice, and the levels of phosphorylated extracellular regulated protein kinase (p-ERK) and hormone-sensitive lipase (p-HSL) were all decreased in *g2e* ^−/−^ mice. Lipolysis is recovered in *g2e* ^−/−^ mice through the addition of protein GIIE. GIIE regulates lipolysis in adipocytes, likely through the ERK/HSL signaling pathway^17^.

Numerous pharmacological approaches have been developed to inhibit sPLA_2_ activity, particularly the hydrolytic activity on the sn-2 position of phospholipid. Indole analogues, first developed by Lilly and Shionogi labs, are the most popular choice because of their strong inhibition effect against sPLA_2_s^2, 11^. For example, LY315920 (Varespladib) strongly inhibits hGIIE (IC_50_ = 0.05 µM), hGX (IC_50_ = 0.075 µM) and hGIIA (IC_50_ = 0.125 µM), and is less potent on hGIB (IC_50_ = 0.75 µM) and hGV (IC_50_ = 0.5 µM)^18^. Consequently, sodium salt of Varespladib (LY315920: Na) has been clinically investigated in severe sepsis, however trials were terminated because of higher mortality rate in the experiment group^19^. Varespladib methyl (Anthera) has been tested in phase three trials for treatment of cardiovascular diseases. Unfortunately it caused adverse effects with increased events of cardiovascular mortality, heart attack, and stroke^20^. These failures may be attributed to the lack of subtype selectivity for varespladib, as it not only inhibits the pro-atherosclerotic effects of GIIA and GV but also inhibits the anti-atherosclerotic effect of GX^21^. Varespladib methyl has also been clinically tested in the treatment of rheumatoid arthritis but was proved ineffective^22^. Me-Indoxam is another indole analogue, with higher potency to hGIIE (0.008 µM) and hGIIA (0.03 µM) than other sPLA_2_s^11^. Based on the co-crystal structure of hGX-Me-Indoxam, a series of indole analogues were designed. Several Me-Indoxam analogues bearing a 4-(2-oxy-ethanoic acid), such as compound 8, 14 and 24 are potent inhibitors for hGIIE (IC_50_ < 0.025 µM), hGIIA (0.33 µM > IC_50_ > 0.025 µM) and hGV (IC_50_ < 0.33 µM)^23^. In summary, many indole analogues have the high potency against hGIIE and hGIIA, but clinical trials have failed mostly due to the lack of selectivity for various sPLA_2_s. Therefore more sPLA_2_ subtype structures are needed to guide the selective inhibitor development. To date, among the 11 mammalian sPLA_2_s, only three protein structures (GIB, GIIA and GX) from various species have been published.

Here we report the crystal structure of hGIIE in its apo form. This will provide structural insight into its enzymatic mechanism and associated biological functions. In addition, we also determined five crystal structures of hGIIE complexed with different indole analogues including compound 8, 14, 24, Me-Indoxam, and LY311727. These will facilitate the structure-based design of selective inhibitors against sPLA_2_s.

## Results

### Overall structures of hGIIE

**Figure 1:**
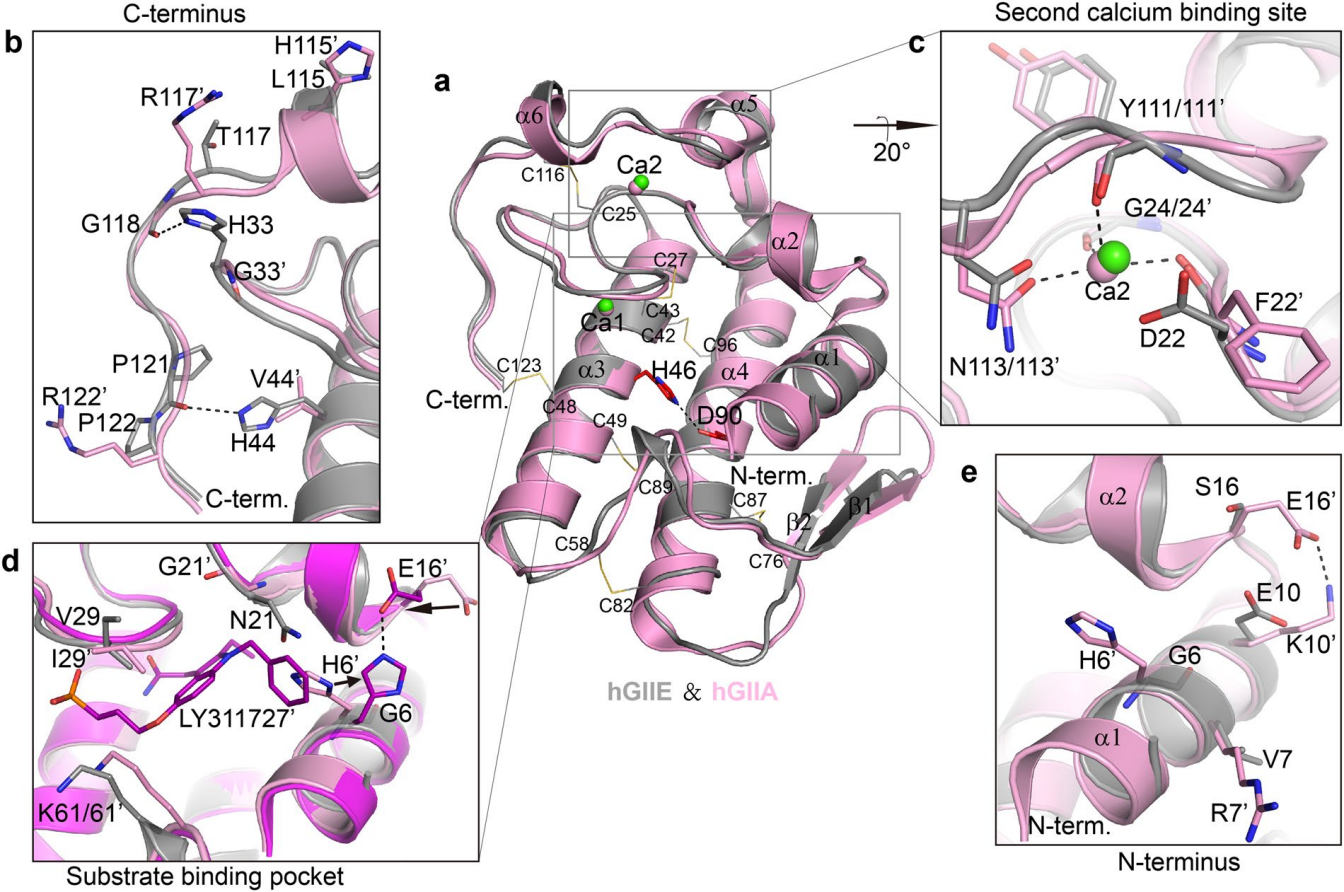
Comparison of hGIIE and hGIIA structures. (**a**) Superposition of the overall structure of hGIIE (gray, apo-hGIIE_1, crystal condition: without CaCl_2_) and hGIIA (pink, PDB: 3U8B), shown in ribbon representation. Secondary elements are numbered with either α (helices) or β (sheets). Catalytic dyad His46 and Asp90 are shown in red sticks. Calcium ions of hGIIE are shown as green spheres. Disulfide bonds are shown in line representation. (**b**) C-terminal region. Basic residues in hGIIA are labeled and shown in stick. Residues of hGIIE in the same place as hGIIA and other essential residues are also labeled and shown in stick. (**c**) The second calcium binding site. Calcium coordination in hGIIA is indicated by black dash lines. (**d**) Superposition of substrate binding pocket of hGIIE (gray), hGIIA (pink), and hGIIA-LY311727 (purple, PDB: 3U8D). Black arrows indicate the positional change of the residues in hGIIA structure upon binding with LY311727. (**e**) N-terminal region. Basic residues in hGIIA are labeled and shown in stick. Residues of hGIIE in the same place are also labeled and shown in stick.

A total of seven crystal structures of hGIIE were obtained, and all of them are in space group P2_1_22 with one molecule in the asymmetric unit (Supplementary Results, Supplementary Table 1). Two are apo-hGIIE structures at the resolution of 2.0 Å and 1.9 Å, with different crystallization conditions: without (apo-hGIIE_1) or with calcium (apo-hGIIE_2). The other five structures are hGIIE with different indole analogues (compound 8, 14, 24, Me-Indoxam, and LY311727). As expected, the protein fold of hGIIE in these structures is similar to previously reported sPLA_2_ structures, including human GIB^24^, GIIA^25^, and GX^26^, with the root-mean squared deviations (RMSD) between them and apo-hGIIE_1 being 1.058 Å, 0.619 Å and 1.062 Å, respectively. Residue dyad His and Asp of the active site and the calcium binding loop (GCXCG) are highly conserved in the sPLA_2_ family (Supplementary Fig. 1). The structure of hGIIE consists of a typical set of three major helices (α1, α3 and α4), two β-strands (β1 and β2), and three short helices (α2, α5 and α6) around the second calcium binding site (Fig. 1a). Similar to hGIIA, hGIIE has seven disulfide bonds.

### Comparison of hGIIE with hGIIA structures

Although hGIIE resembles hGIIA with 55% sequential identity and similar protein fold, it still presents some subtle differences. From the overlay of hGIIA and hGIIE structure, the apparent difference can be found in the substrate binding pocket, second calcium binding site, C-terminus, the loop between β1 and β2 (β1/β2 loop), and the surface electrostatic charge distribution (Fig. 1 and Supplementary Fig. 2d).

The substrate binding pocket is formed by the α1, α2, the typical calcium binding loop and the loop region around Lys61 (Fig. 1d). For hGIIA, there is a high content of basic residues in α1, including His6, Arg7 and Lys10. Residue Glu16 forms a salt bridge with Lys10 in the apo-hGIIA structure (Fig. 1e). His6 swings out of the substrate binding pocket and forms a hydrogen bond with Glu16 in the complex structure of hGIIA with LY311727 (Fig. 1d). In contrast, in hGIIE structure, α1 is relatively neutral or negatively charged, with residue Gly6, Val7 and Glu10 (Fig. 1e). In this pocket, besides the difference in α1, the loop region around Lys61 in hGIIE is further away from the N terminus, compared with hGIIA (Fig. 1d). Lys61 in hGIIA is located directly opposite to Val29, and the two of them form the bridge-like cap above the pocket (Fig. 1d). However, Lys61 in hGIIE is facing Ile29, and hGIIE presents a much wider entrance (Fig. 1d). In addition, for hGIIE, the side chain of Asn21 in α2 participates in the formation of the substrate binding pocket (Fig. 1d).

At the C-terminal region, hGIIA has a patch of basic residues, including His115, Arg117 and Arg122 (Fig. 1a). On the other hand, hGIIE has a neutral C-terminus with residues Leu115, Thr117 and Pro122. In the C terminus of hGIIE, carbonyl oxygen of Gly118 and Pro121 form hydrogen bonds with His33 and His44, respectively (Fig. 1a). His33 is the unique residue in hGIIE, while in other human sPLA_2_s there is Gly at that position (Supplementary Fig. 1). Additionally, hGIIA has an extensively positively charged interfacial binding surface (i-surface) (Supplementary Fig. 2c) as reported^26^. In contrast, hGIIE presents an i-surface with much less positive charge (Supplementary Fig. 2a).

Interestingly, a unique hydrophobic region is found on the back of the i-surface near the C-terminal region of hGIIE. There is a hydrophobic core formed by Trp34, Trp41, Pro35 and Pro121 (Supplementary Fig. 2b). Trp34 is unique in hGIIE, whereas other sPLA_2_s have polar residue in this position (Ser34 in hGIIA). Trp41 forms an edge-to-face π-π interaction with His44. Trp41 is conserved in human sPLA_2_s, except in hGIB and hGIIA, which have basic residue Lys and Arg, respectively instead (Supplementary Fig. 1).

In hGIIE structure, the β1/β2 loop moves away from the α1 helix, and the distance between Lys11 in the α1 helix and Glu71 in the β1/β2 loop is 11 Å (Supplementary Fig. 2e), compared with other reported human sPLA_2_s (Supplementary Fig. 2d). This distance for hGIIA is about 4.8 Å. Residues from 71 to 74 in this loop vary in different kinds of sPLA_2_ members (Supplementary Fig. 1).

### The typical calcium binding site in hGIIE structures

**Figure 2:**
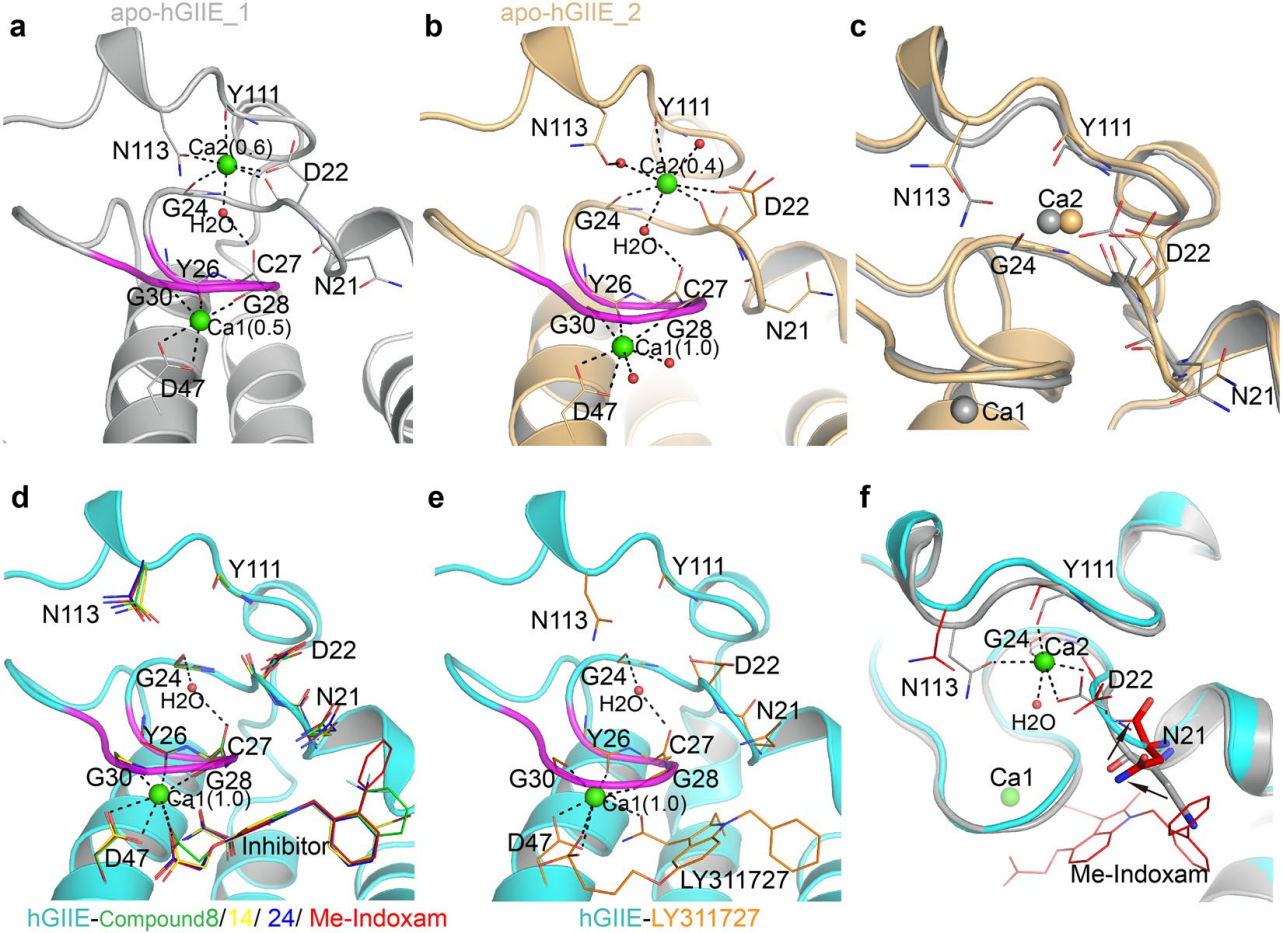
Analysis of the calcium binding sites of hGIIE. Residues involved in the calcium binding sites are labeled and shown in line presentation. Water molecules are shown in red spheres, calcium ions are shown in spheres, and occupancy of calcium is labeled in parenthesis. Inhibitors are shown in line representation. (**a**) Calcium binding sites of apo-hGIIE_1 from the crystal formed in the condition without calcium ion. (**b**) Calcium binding sites of apo-hGIIE_2 from the crystal formed in the presence with 10 mM CaCl_2._ Asp22 has two alternative conformations, colored in orange and light orange. (**c**) Superposition of the second calcium binding site of apo-hGIIE_1 (gray) and of apo-hGIIE_2 (light orange). (**d**) Calcium binding sites of the inhibited hGIIE structures with compound 8 (green), 14 (yellow), 24 (blue) and Me-Indoxam (red). (**e**) Calcium binding sites of the inhibited hGIIE structure with LY311727. (**f**) Superposition of calcium binding sites of apo-hGIIE_1 (gray) and hGIIE-Me-Indoxam (cyan). Asn21 is shown in stick presentation. Black arrows indicate the positional change of the residues of hGIIE after binding with Me-Indoxam. There is no change in terms of disulfide bonds between the two apo hGIIE structures, therefore, the disulfide bonds were not labeled in this figure for clarity reason.

Calcium in the typical calcium binding loop is essential for the binding and catalysis of substrate^27^, and calcium in this site (Ca1) is observed in all hGIIE structures (Fig. 2). In every structure, the calcium is coordinated by carbonyl oxygen from Tyr26, Gly28 and Gly30, and two carboxyl oxygen of Asp47 (Fig. 2a,b,d and e). In the apo-hGIIE_1 structure, crystallized in the condition without calcium, the Ca1 is surprisingly not fully occupied, with only 54% occupancy in this typical calcium binding site (Fig. 2a). However, in the hGIIE structures with compound 8, 14, 24 and Me-Indoxam, all crystallized from conditions without calcium, Ca1 is seven coordinated with 100% occupancy, with the extra two coordination from the carboxyl oxygen and amide oxygen in inhibitors (Fig. 2d). In the hGIIE-LY311727 complex structure, Ca1 is 100% occupied, with the extra two coordination from the amide oxygen and phosphate oxygen in LY311727 (Fig. 2e). In the apo-hGIIE_2 structure, crystallized in the presence with 10 mM CaCl_2_, Ca1 with 100% occupancy is seven-coordinated, and two water molecules provide the extra two coordination (Fig. 2b).

### The flexible second calcium binding site in hGIIE structures

Similar with hGIIA, hGIIE also has the second calcium binding site (Fig. 1c). However, calcium in the second binding site (Ca2) seems unstable in the hGIIE structures. In the apo-hGIIE_1 structure, the occupancy for Ca2 is 57%, which is six-coordinated by the carbonyl oxygen of Asp22, Gly24, Tyr111, carboxyl oxygen of Asp22, amide oxygen of Asn113, and one water molecule (Fig. 2a). In the apo-hGIIE_2 structure, Ca2 is 39% occupied and coordinated by carbonyl oxygen of Gly24, Tyr111, three water molecules, and either the carbonyl or carboxyl oxygen of Asp22 (Fig. 2b). In all inhibitor bound hGIIE structures, to our surprise, the Ca2 binding site is not occupied (Fig. 2d and e). In these structures, residues that formed the second calcium binding site, including Asp22, Gly24, Tyr111 and Asn113, expand slightly without the stabilization of the second calcium (Fig. 2f). Adjacent to the second calcium binding site, the main chain of Asn21 has a flip of about 172°, and the side chain of Asn21 swings away from the substrate binding pocket, in order to accommodate the inhibitors (Fig. 2d,e and f).

### The calcium binding sites are essential for enzymatic activity of hGIIE

To assess the contribution of calcium to the enzymatic activity of hGIIE, we performed site-directed mutagenesis in the two calcium binding sites of hGIIE and measured the enzymatic activity and kinetic properties (K_cat_/K_m_) of these mutants using sPLA_2_ enzyme assay. The enzymatic activity of wild type (WT) hGIIE is 3.798 U/mg, comparable to previously reported hGIIE activity^28^. The catalytic efficiency value (K_cat_/K_m_) of WT hGIIE is 34.01/mM/s.

**Figure 3:**
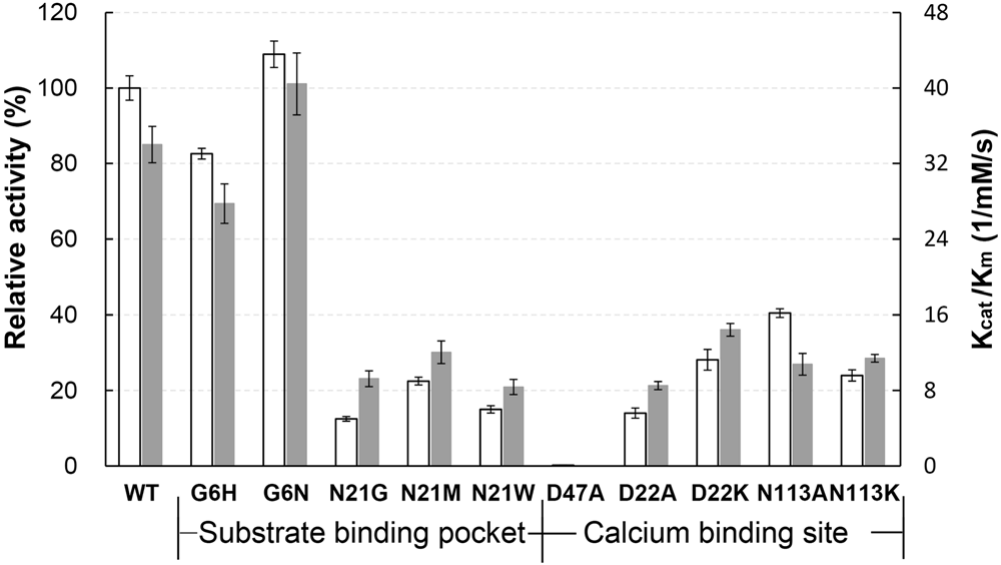
Enzymatic activity and kinetic parameters of wild type and mutant hGIIE. Enzymatic activity of WT (3.80 ± 0.12 U/mg) was defined as 100% relative activity. Data is shown as the means values ± s.d. (n = 3) (Data details are shown in Supplementary Table 2).

To confirm the significance of the first calcium binding, residue Asp47, which is the only residue stabilizing the calcium with side chain, was mutated to Ala. As expected, the D47A mutant retains only 0.19% enzymatic activity (Fig. 3).

To study the function of the second calcium binding, we performed mutational study for residue 22 and 113. Sequence alignment shows that residues in the second calcium binding site are not conserved in the human sPLA_2_ family, especially in position 22 and 113 (Supplementary Fig. 1). hGIB^24^ and hGX^26^ have no second calcium binding site. In hGX, the charge repulsion from cationic residue Lys22 renders the calcium binding at this region not favorable (Supplementary Fig. 3). In hGIIE, mutants D22K and N113K retained only 28.14% and 23.98% enzymatic activity respectively, with decreased catalytic efficiency (Fig. 3), suggesting that the second calcium is crucial to the enzymatic activity for hGIIE. Similarly, mutant D22A and N113A have decreased enzymatic activity compared to WT hGIIE.

Residue in position 21, adjacent to the second calcium binding site, is not conserved in the human sPLA_2_ family (Fig. 1). To study the role of Asn21 in the enzymatic activity of hGIIE, we designed hGIIE variants, including N21G (mimicking hGIIA and hGV), N21M (mimicking hGX) and N21W (mimicking hGIID). These mutants showed 78–88% loss of enzymatic activity and significantly decreased catalytic efficiency than WT (Fig. 3).

### Phospholipid head group selectivity of hGIIE

**Figure 4:**
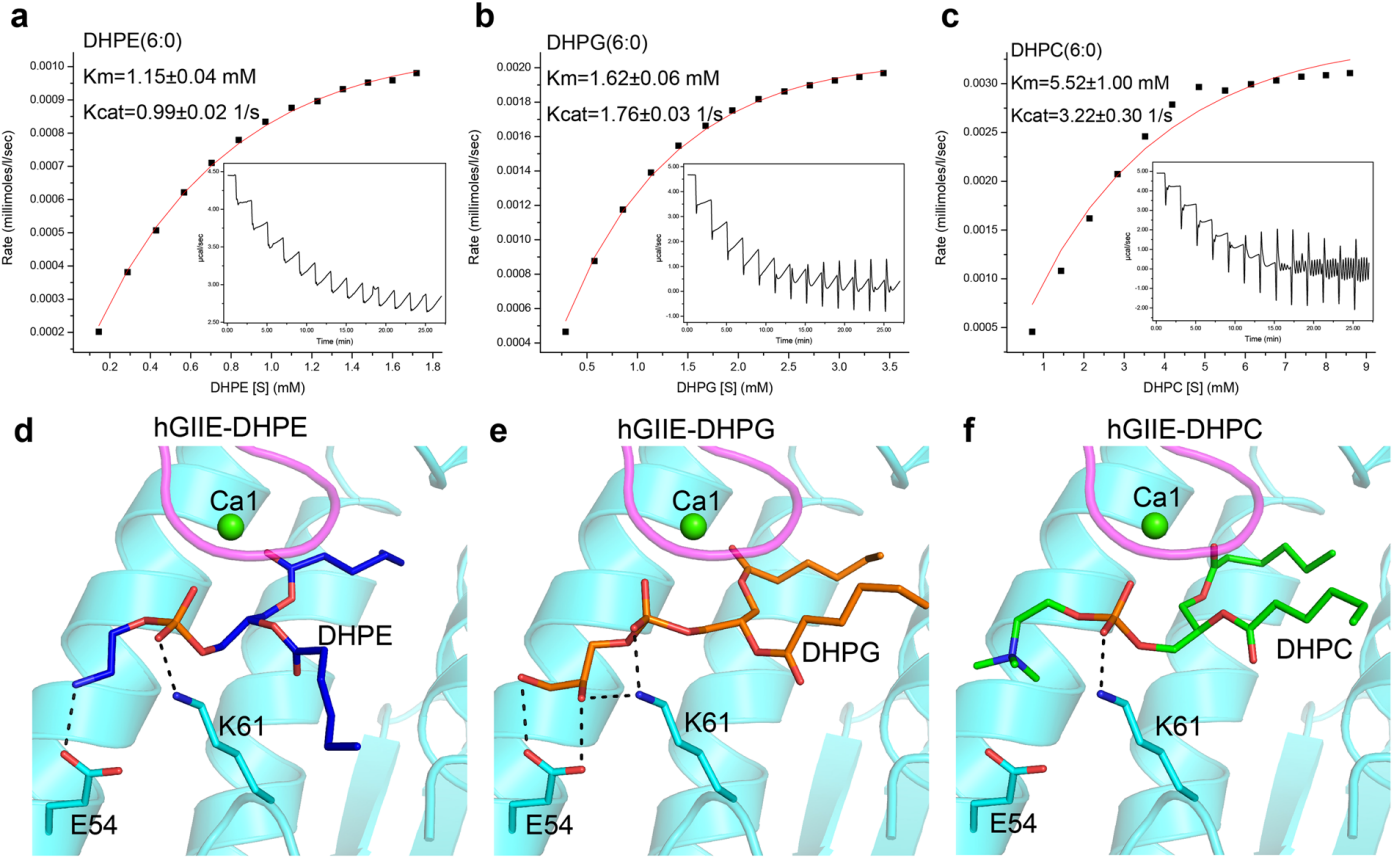
Phospholipid head group selectivity of hGIIE. (**a–c**) Enzymatic activity of hGIIE determined by isothermal titration calorimetry method: (**a**) 1, 2-dihexanoyl-sn-glycero-3- phosphor-ethanolamine (DHPE); (**b**) 1, 2-dihexanoyl-sn-glycero-3-phosphor-rac-1-glycerol (DHPG); (**c**) 1, 2-dihexanoyl-sn-glycero-3-phosphocholine (DHPC). Heat flows are converted to specific enzyme activities. The raw titration curves are shown in the inserts. Data is fitted to the standard Michaelis-Menten equation, trend line is shown in red. (**d–f**) Docking model of hGIIE with the phospholipid: (**d**) hGIIE-DHPE; (**e**) hGIIE-DHPG; (**f**) hGIIE-DHPC. Hydrogen bond interaction is indicated by black dash lines, and residues involved are shown as stick.

We determined the specific activities of hGIIE on phospholipids with different head groups using the isothermal titration calorimetry method. hGIIE presents low substrate preference towards those phospholipids in mM scale. Among them, hGIIE has the highest affinity (the lowest K_m_ value) but lowest turn-over number towards 1, 2-dihexanoyl-sn-glycero-3- phosphor-ethanolamine (DHPE) (Fig. 4a). To anionic 1, 2-dihexanoyl-sn-glycero-3-phosphocholine (DHPG), hGIIE has no preference with the medium catalytic turn-over number and medium K_m_ value (Fig. 4b). hGIIE shows the lowest affinity (highest K_m_ value) but highest turn-over number to 1, 2-dihexanoyl-sn-glycero-3-phosphocholine (DHPC) (Fig. 4c).

To gain understanding of this substrate selectivity, DHPE, DHPG and DHPC were docked onto the active site of hGIIE, respectively. All of the phosphate in the head group of the three substrates can form a salt bridge with residue Lys61 (Fig. 4d–f). DHPE, which has the highest affinity to hGIIE, forms another hydrogen bond with Glu54 by the amide nitrogen in the head group (Fig. 4d). Two hydroxyl oxygen atoms in the head group of DHPG also can form hydrogen bonds with Glu54, and one hydroxyl oxygen forms a hydrogen bond with Lys61 (Fig. 4e). Unsurprisingly, the head group of DHPC cannot form any additional interaction with hGIIE (Fig. 4f), partially explaining the lowest affinity against DHPC for hGIIE.

### Structural basis of inhibitor recognition by hGIIE

**Figure 5:**
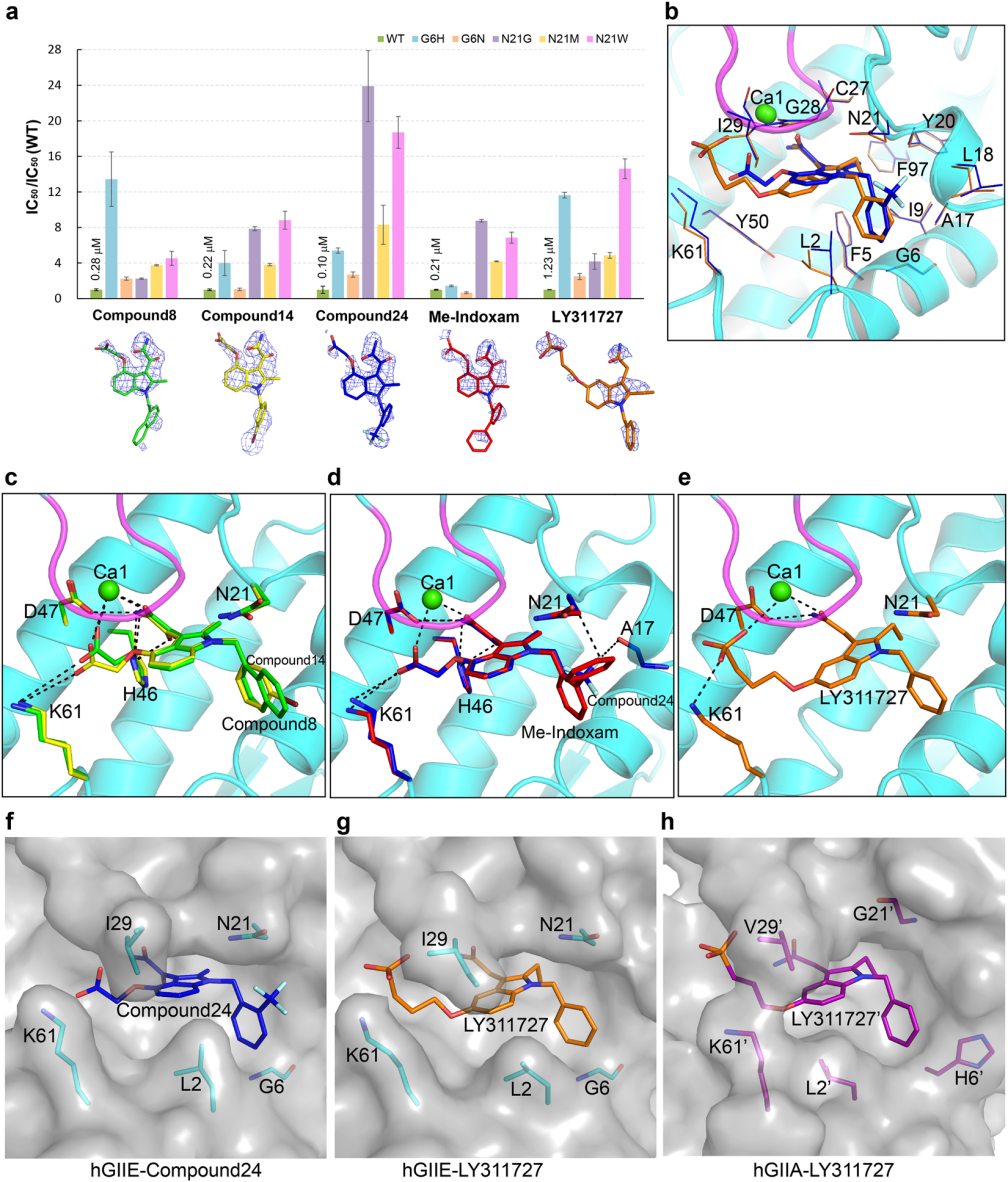
Analysis of complex hGIIE structures with different compounds and inhibiton data. (**a**) Inhibition data against WT and mutated hGIIE by LY311727, compound 8, 14, 24, and Me-indoxam. IC_50_ values for WT are labeled. Inhibition data against the mutant is shown as IC_50_ (mutant)/IC_50_ (WT). Data is shown as the means values ± s.d. (n = 2) (Data details are shown in Supplementary Table. 3). Fo-Fc electron density map at 3 σ level is contoured around the compounds. (**b**) Hydrophobic interaction between protein hGIIE and compound. Residues involved in the interaction between hGIIE and LY311727 (orange stick) and compound 24 (blue stick) are labeled and shown in line representation. (**c–e**) Hydrogen bond interaction between hGIIE and compounds, and interactions between calcium and compounds: (**c**) Superposition of hGIIE-compound 8 (green) and hGIIE-compound 14 (yellow), (**d**) Superposition of hGIIE-compound 24 (blue) and hGIIE-Me-Indoxam (red), (**e**) hGIIE-LY311727 (orange). Hydrogen bond interaction and calcuim interaction are indicated by black dash lines. Residues involved are shown as stick. (**f–h**) Surface presentation of the substrate binding pocket: (**f**) hGIIE-compound 24 (blue), (**g**) hGIIE-LY311727 (orange), (**h**) hGIIA-LY311727 (purple), main residues are shown as sticks.

Previous reports showed that compound 8, 14, 24, and Me-Indoxam have best inhibition effect against hGIIE when compared with other sPLA_2_s^23^. These compounds have the same indole rings bearing a 2-methyl group, a 3-glyoxamide side chain and a 4-(2-oxy-ethanoic acid) side chain, and have varying substituents at the N1 position. In the N1 position, compound 8 has 2-napthalene-CH_2_ group, compound 14 has 3-Br-benzene-CH_2_ group, compound 24 has 2-CF_3_-benzene-CH_2_ group, and Me-Indoxam has 2-benzene-benzene-CH_2_ group. Among these, compound 24 is the most potent inhibitor (IC_50_ = 0.10 µM) against WT hGIIE, and the other three have similar IC_50_ ranging from 0.21 to 0.28 µM (Fig. 5a).

To identify the essential residues in hGIIE responsible for the inhibition effect of these compounds, we obtained complex crystal structures of hGIIE with these inhibitors. The electron density is unambiguous for each of the compounds (Fig. 5a). In all four complex structures, the 3-glyoxamide side chain and 4-(2-oxy-ethanoic acid) side chain in indole rings of these compounds can form hydrogen bonds with the active site residues and interact with the calcium at Ca1 site (Fig. 5c,d). Amide nitrogen in 3-glyoxamide side chain form hydrogen bonds with the active site His46 and Asp47. An additional hydrogen bond exists between carbonyl oxygen in the 3-glyoxamide side and His46. A salt bridge is formed between the carboxyl group in the 4-(2-oxy-ethanoic acid) side chain and Lys61. Amide oxygen in the 3-side chain and one of the carboxyl oxygen in the 4-side chain of inhibitors are coordinated with calcium instead of water in the apo-hGIIE_2 structure (Fig. 3b). Indole rings of these compounds form π-NH interaction with amide nitrogen of Asn21.

Compared with compound 8 and 14, compound 24 can form additional interactions with residue Ala17 and Asn21 using the trifluoromethyl group (Fig. 5d). Me-Indoxam can form an amide-π interaction with the amide group of Asn21 (Fig. 5d). All of these compounds show decreased inhibition effect in varying degrees, about 2–24 fold, against mutants in Asn21 (N21G, N21M and N21W) (Fig. 5a). Therefore, Asn21 in hGIIE is one critical residue contributed to the best effect of these indole analogues against hGIIE.

LY311727 is another class of indole analogue inhibitor, which has the indole ring bearing a 2-ethyl group, a 3-acetamide group, and 5-oxypropylphosphate (Fig. 5a). Phosphate oxygen and amide oxygen of LY311727 coordinates with calcium, and amide nitrogen forms a hydrogen bond with Asp47 (Fig. 5e). The indole ring of LY311727 also forms π-NH interaction with amide nitrogen of Asn21. LY311727 shows decreased inhibition effect in varying degrees, about 4–15 fold, against mutants in Asn21 (N21G, N21M and N21W) (Fig. 5a).

Unlike the other four compounds mentioned above, LY311727, lacking carbonyl group in 3-modifed group of indole ring, lost the interaction with His46 (Fig. 5e). LY311727 has 2-ethyl group instead of methyl, which renders the indole ring of LY311727 incapable of burying deeply into the hydrophobic pocket (Fig. 5b). In the N1 position, lacking the hydrophobic interaction with Leu18, the unmodified benzene group of LY311727 weakens the surface complementarity between inhibitor and hGIIE (Fig. 5b). These differences partially explain the 4–12 fold decreased inhibition effect against WT hGIIE for LY311727, compared with compound 8, 14, 24 and Me-Indoxam (Fig. 5a).

These five compounds all sit in the hydrophobic pocket of hGIIE formed by residue Leu2, Phe5, Gly6, Ile9, Ala17, Leu18, Tyr20, Asn21, Cys27, Gly28, Ile29, K61, Tyr50 and Phe97 (Fig. 5b). From the sequence alignment of sPLA_2_s, residue Phe5 and Phe97 are conserved in hGIIE and hGIIA. However, they are both replaced by Leu in hGV and hGX (Supplementary Fig. 1). Phe5 and Phe97 were supposed to be the critical residues contributing to the high affinity of indole analogues with hGIIA and GIIE^26, 29^. Here we designed the single and double mutations for residues Phe5 and Phe97 to smaller residue Leucine. However, single mutant F5L, F97L, and double mutant F5L_F97L were inhibited in the same order of magnitude by these compounds as WT (Supplementary Fig. 5).

## Discussion

Similar with other sPLA_2_s, hGIIE structure presents a conserved active site and calcium binding loop. The difference between hGIIE and other sPLA_2_s could provide insights into understanding the specific roles hGIIE played in the catalytic reaction and other biological processes.

Anionic PG is the preferred substrate for most sPLA_2_s, especially for hGIIA. However, hGIIE, sharing highest sequence similarity with hGIIA, has no preference for PG^11^. The positively charged residues in the N-terminus and C-terminus of hGIIA are favorable for the PG substrate binding. As reported, charge-reversal double mutant in residue R7 and K10 of hGIIA has 45-fold loss in the affinity to PG. Similarly, mutants in basic residues in the C-terminus showed a significant decreased affinity to PG, indicating the involvement of these residues in the binding to anionic liposomes^30^. In terms of hGIIE, the lower activity to PG should be attributed to the neutral residues in the N-terminal and C-terminal region of hGIIE.

Recent studies for GIIE-knockout mice revealed the non-redundant role of hGIIE in diverse biological events. GIIE appears to release LPE selectively to regulate the mouse skin homeostasis^14^. In lipoprotein particles, PC is a dominant phospholipid component. However, GIIE preferentially hydrolyzed PE and PS, which are present at trace level^31^. Likewise, our *in vitro* enzymatic study also showed that GIIE has higher affinity to PE than PC (Fig. 4a,c). As shown in the substrate binding model of hGIIE, both the head group of PE (Fig. 4d) and PS (Supplementary Fig. 6), but not the head group of PC (Fig. 4f), can form additional hydrogen bonds with Glu54. Glu54 appears to be important for the selectivity of phospholipids in the biological process of hGIIE.

Some biological functions of sPLA_2_s have been shown to be independent of their enzymatic activity, thus indicating that hGIIE may function through binding to some receptors^5^. The hydrophobic C-terminal region of hGIIE, along with the adjacent hydrophobic core formed by Trp34, Trp41, His44, Pro35 and Pro121 (Supplementary Fig. 2b), may serve as the potential receptor or lipid binding domain. Further study is needed to uncover the functional roles this region of hGIIE played.

The functional implication for the calcium in the second binding site of hGIIE is intriguing. As reported for GIIA, the second calcium may play the role of a supplemental electrophile by stabilizing the oxyanion of the tetrahedral intermediate through a hyper-polarization of the peptide bond between Cys27 and Gly28^25^. Similarly in apo-hGIIE structures, a water molecule, part of the second calcium hydration shell, forms a hydrogen bond to the carbonyl oxygen of Cys27 and links the second calcium to the oxyanion (Fig. 3a,b and Supplementary Fig. 7a). Mutational experiments in the second calcium binding site further support this “supplemental electrophile” mechanism. An interesting observation for the calcium binding in these hGIIE structures is the occupancy for that first and second calcium binding site. In the inhibitor bound structures, occupancy of Ca1 rose to 100% and the second calcium disappeared. In the apo-hGIIE_1 structure, Ca1 is only 54% occupied and the occupancy for Ca2 is 57%. This raises a possibility that calcium in the second binding site could move to the first calcium binding site when needed. Therefore, hGIIE may have a cost-effective way to use calcium, and Ca2 can act as backup to support the partially occupied Ca1. The second calcium binding site of hGIIE is unstable with a flexible region around Asp22 and Asn113 (Fig. 3c). This may favor the release of the second calcium. Besides hGIIE, in the previously reported structures of mammalian sPLA_2_, only GIIA has the second calcium binding site^32^. However, the second Ca of hGIIA is strongly coordinated by residue Phe22, Gly24, Tyr111 and Asn113 (Fig. 1c). So the backup function of the second calcium may be a unique feature for hGIIE. In summary, calcium in the second binding site of hGIIE may act as the supplemental electrophile for oxyanion and also as the backup for Ca1.

Compared to WT hGIIE, mutants in Asn21 present dramatically decreased enzymatic activity (Fig. 4). Asn21, which forms the upper boundary for the substrate binding channel of hGIIE (Fig. 5f,g), may play an important role in the phospholipid substrate binding to the pocket. In order to accommodate the inhibitor, carbonyl oxygen in the main chain of Asn21 was flipped around 172° in all inhibitor bound hGIIE structures (Fig. 3f). This change induced by inhibitors is inevitable; otherwise Asn21 would clash with inhibitors (Fig. 3f). Among other human sPLA_2_ members, only hGIB (PDB: 3ELO) has residue Asn21, and presents the same main chain conformation as in the inhibitor-bound hGIIE structures (Supplementary Fig. 3). If the role of Asn21 can be applied to the substrate catalysis action of hGIIE, conformation change of Asn21 would induce the side chain swing of Asp22. The flexibility of Asp22 would support the notion that Ca2 serves as the backup for Ca1 as discussed earlier.

Compound 8, 14, 24 and Me-Indoxam have various inhibition effects toward different members of sPLA_2_s (22). Two non-conserved regions of sPLA_2_s participate in the interaction with inhibitors: resiude17–22 and N-terminal helix.

Residues from 17 to 22, especially residue 21 may interact or clash with the 2-modified benzene group in the N1 position of these indole analogues. For hGIIE, compound 24 and Me-Indoxam, which have 2-modified benzene group in the N1 position of indole, form the additional interaction with Asn21 (Fig. 5d). Therefore, these compounds, especially compound 24, have the best inhibitory effect against hGIIE compared to other sPLA_2_s. For hGIID, the large hydrophobic residue Trp21 may clash with the 2-modified benzene group in the N1 position of indole, so compound 24 has over 100-fold decreased effect against hGIID in comparison to hGIIE^23^. Similar to hGIID, Met21 in hGX would cause the steric hindrance with the 2-modified benzene group in the N1 position of indole, resulting in compound 24 having about 50-fold decreased effect against hGX than hGIIE^23^. In this study, compared with WT hGIIE, these compounds present 4–8 fold decreased inhibition effect against mutant N21M, and 5–19 fold decreased inhibition effect against mutant N21W (Fig. 5a). For hGIIA, Gly21 can neither form the hydrogen bond nor clash with these compounds (Fig. 5h). The IC_50_ value of these compounds against hGIIA is higher than hGIIE^23^. For hGIIE, these compounds present 2–24 fold decreased inhibition effect against mutant N21G compared to the WT hGIIE (Fig. 5a). Our results on the co-crystal structure of hGIIE N21G mutant with compound 24 also confirm that the inhibitor binds much weaker to the mutant protein than the WT protein. Compound 24 in N21G structure has poor Fo-Fc map compared to the WT structure (Supplementary Fig. 9), and the occupancy of compound 24 in N21G structure is refined to only 68%.

Residues in the N-terminus, which form part of the entrance of the binding pocket of sPLA_2_, may participate in the interactions with the 3-modified benzene group in the N1 position of these indole analogues (Fig. 5c). hGIIA has a unique His6 acting as the door of the pocket. In the LY311727-hGIIA complex structure, the entrance is open as in the hGIIA-transition state analogue (TSA) complex structure^25^ (Fig. 1d). In this study, compound 8 and 14, with 3-modified group in the N1 position of indole ring, have 4–13 folds decreased effect against hGIIE mutant G6H compared to the WT (Fig. 5a), possibly due to the entrance blockage from His6. In conclusion, besides Asn21, Gly6 in hGIIE is another essential residue that contributes to the inhibition effects of these indole analogues against hGIIE.

hGIIA has been generally regarded as the drug target for decades, and indole analogues mentioned in this report have a slightly higher inhibition effect against hGIIE than hGIIA^23^. Therefore, subtype selective drugs that can only inhibit hGIIA but not hGIIE should be developed. From the difference of the binding pocket of hGIIE and hGIIA (Fig. 5f,g,h), several notions could be suggested for the design of selective inhibitors of hGIIA based on the indole analogue bearing 2-methyl group, 3-glyoxamide side chain and 4-(2-oxy-ethanoic acid) side chain. Firstly, H6 in hGIIA forms the right wall of the pocket (Fig. 5h), whereas hGIIE has no barrier on the right side of the pocket (Fig. 5g,h). To design the selective inhibitor of hGIIA, 3-modified benzene group, especially the bulky group in the N1 position of indole, should be avoided. Secondly, selecting the compound that can cause the steric clash with Asn21 in hGIIE but not Gly21 in hGIIA is another choice. Thirdly, Lys61 in hGIIE is located further away from Leu2, and an additional channel becomes available between them in hGIIE (Fig. 5f,g). Therefore, modification in the 6- or 7- position of the indole ring would induce steric clash with Leu2 in hGIIE but not Leu2 in hGIIA. Finally, Lys61 in hGIIA is located directly opposite to Val29, and two of them form a cap above the pocket (Fig. 5h). However Lys61 in hGIIE is facing Ile29, and hGIIE presents a much wider entrance, so hGIIE but not hGIIA can accommodate a bigger group in this position.

With a wide range of biological functions of sPLA_2_ isoforms uncovered, it is imperative to obtain more structural information for different subtype sPLA_2_s to facilitate their functional studies, and to help the subtype selective inhibitor development. In the present study of apo-hGIIE structure, its flexible second calcium binding site and the region around residue Asn21 were proved to be essential for its enzymatic activity. Complex structures for inhibitor bound hGIIE, coupled with the mutagenesis analysis, revealed the key residues of hGIIE that form interaction with inhibitors, and elucidated the difference in the inhibitor binding pocket with other sPLA_2_s. This will provide key structure insights for the design of selective inhibitors of sPLA_2_s.

## Methods

### Chemicals

PrimeStar DNA polymerase and restriction enzymes were purchased from Takara. sPLA_2_ Assay kit was purchased from Cayman Chemicals. 1, 2-dihexanoyl-sn-glycero-3-phosphor-rac-1-glycerol (DHPG), 1, 2-dihexanoyl-sn-glycero-3-phosphocholine (DHPC), 1, 2-dihexanoyl-sn-glycero-3-phosphoethanolamine (DHPE) were purchased from Avanti. LY311727 was purchased from Sigma-aldrich as 95% purity. Compound 8, 14, 24, and Me-Indoxam were synthesized according to the literature^23^.

### Plasmid construction

The gene encoding for hGIIE protein without signal peptide was amplified by PCR using the template of recombinant vector pET21a-hGIIE (cloned from cDNA encoding full-length of hGIIE, Genbank/EMBL Accession NM_014589) from our laboratory. The PCR products were purified using DNA extraction kit and cloned into vector pGAPZαA (Expression vector, Invitrogen) between EcoRI and XbaI restriction sites. The ligation mix was transformed into *Escherichia coli* TOP 10, positive clones were screened by 40 µg/mL Zeocin (Invitrogen) and verified by PCR and DNA sequencing. Mutagenesis was carried out by the overlap extension PCR^33^ using the recombinant plasmid pGAPZαA _hGIIE as the template. All the primers are shown in Supplementary Table 4.

### Protein production and purification

The expression plasmid pGAPZαA _hGIIE was linearized with BlnI. *Pichia pastoris* × 33 cells (Invitrogen) were prepared according the Pichia expression manual and transformed with 2 µg of linearized plasmids by electroporation with Gene Pulser apparatus (Bio-rad) at 1000 V using a 0.2 cm cuvette. Transformants were screened on YPD plates with 100 µg/mL Zeocin. After incubation for 48 h at 29 °C, positive clones were further selected by PCR and directly transferred to 10 mL YPD medium by shaking at 200 rpm, 29 °C for 3 days. The clone with the highest expression was selected based on results of SDS-PAGE. To scale up, the selected strain was cultured in 400 mL shake flask with YPD medium for 48 h as the seed. Then fermentation was conducted in 14 L bioreactor (Eppendorf-NBS) by fed-batch cultivation, with the temperature controlled at 29 °C, pH at 5.8, and aeration rate above 30% dissolved oxygen (DO). pH was controlled by automatic addition of 30% NH_4_OH and agitation speed was automatically controlled at a set point of 30% DO. The initial culture medium with 5 L volume contained the basal salt medium and trace element solution^34^. 2 L feeding medium, which contained the same element as initial medium, was fed into bioreactor with the flow speed at 2 mL/min after the culture started for 12 h. 10 mL samples were fetched out every 6 h during fermentation, and yield of protein was monitored by SDS-PAGE analysis of supernatant. After 55 h of fermentation, the culture broth was centrifuged at 12,000 × g for 15 min at 4 °C to remove the cells.

Culture supernatant was concentrated 5 times using vivaflow 200 system (Sartorius), and then diluted 4 times with buffer (10 mM MES pH 6.0). Purification was achieved by SP HF (GE Healthcare) column and then by Superdex 75 (GE Healthcare).

### Crystallization and data collection

Purified protein sample was concentrated to the final concentration of 3.2 mg/mL in 20 mM Tris-HCl, 200 mM NaCl, pH 8.8 without or with 10 mM CaCl_2_ by ultrafiltration (Millipore Amicon). Initial crystallization was screened by vapor diffusion method using sparse-matrix crystallization screening kits (Hampton Research) at 4 °C. Protein sample was mixed with equal volumes of precipitant in 0.6 µL hanging drop on 96-well microplate (Grenier) using mosquito (ttplabtech). Crystals appeared under the condition of 0.1 M Bis-Tris propane, 2 M NaCl, pH 7 after 1 week.

Before data collection, Crystal was soaked into cryoprotectant contained 10% glycerol and then flash cooled in liquid nitrogen. X-ray diffraction data for apo-hGIIE_1 crystal (formed in the condition without calcium) was collected at the wavelength of 0.9793 Å using synchrotron radiation on beamline BL17U1 at Shanghai Synchrotron Radiation Facility at 100 K. Protein complex structures with compound 8, 14, 24, Me-Indoxam, and LY311727 (Sigma-aldrich) were obtained respectively by soaking the apo-hGIIE_1 crystal into cryoprotectant containing 3 mM inhibitor for 20 min. X-ray diffraction data for soaked crystals and apo-hGIIE_2 crystal (formed in the presence of calcium) were collected at the wavelength of 1.5418 Å using Oxford Diffraction GeminiR Ultra system respectively at 100 K.

### Structure determination and refinement

Diffraction data were indexed and integrated by MOSFLM^35^, then scaled by Aimless^36^ from CCP4 package^37^. For diffraction data of apo-hGIIE_1 crystal, 5% data were randomly selected for the FreeR calculation. As all data sets share very similar cell parameters, for successful cross validation, the test set selection for FreeR calculation of all later data sets was copied from the first data set and preserved among all data sets. The initial structure was obtained by molecular replacement^38^ using human sPLA_2_ GIIA structure (PDB: 1POE). Refinement was carried out using REFMAC5^39^. Coot^40^ was used to build and fit models into electron density. Occupancy of calcium ion was refined using phenix refinement^41^. The stereochemistry of the structures were checked using MOLPROBITY^42^. Ray traced images were generated using PyMOL^43^. Refinement statistics for each final model are recorded in Supplementary Table 1.

### Enzymatic Kinetics of wild type and mutated hGIIE

Enzymatic Kinetics of hGIIE assay was carried out using sPLA_2_ Assay kit (Cayman, Ann Arbor, MI, USA). Substrate of this assay is 1, 2-Diheptanoyl Thio-phosphatidylcholine (DTPC). After hydrolysis of the thio ester bond in the sn-2 position by phospholipase A_2_, free thiols are detected using 5, 5-dithio-bis-nitrobenzoic acid (DTNB). Activity assay was carried out on a 96-well plate at 25 °C. 10 µL DTNB, 10 µL protein sample and 5 µL buffer (25 mM Tris-HCl, pH 7.5, 10 mM CaCl_2_, 100 mM KCl, 0.3 mM Triton X-100) were pooled in the well, then the reaction was initiated by adding 200 µL substrate solution to the well simultaneously. Absorbance at 414 nm was measured every 1 min for 15 min. Different final concentration of substrate DTPC were used, including 0.083 mM, 0.166 mM, 0.322 mM, 0.415 mM, 0.83 mM, 1.245 mM and 1.66 mM. Protein concentration was adjusted to ensure the absorbance increase of 0.01 to 0.1 per minute. Curve was fitted to Michaelis-Menten equation and Lineweaver-Burk plot for the estimation of K_m_ and V_max_ using GraphPad Prism 5, K_cat_ was calculated by V_max_/enzyme concentration.

### Isothermal Titration Calorimetry (ITC) method to determine phospholipid head group specificity of hGIIE

Reactions were performed with Microcal ITC200 (GE Healthcare) at 25 °C, and data were collected and analyzed by ORIGIN. A multiple injection kinetic assay was conducted using a stirring speed of 750 rpm. 2 µM protein hGIIE (200 µL) in the reaction cell was titrated with 13 × 3 µL injection of phospholipid (10–50 mM) using a 120 s delay between successive injections. The apparent enthalpy (ΔH) for the hydrolysis of phospholipid by hGIIE was determined in a separate experiment in which 1 × 6 µL injections of phospholipid into 2 µM protein hGIIE (200 µL) was made using spacing 1800 s. Buffer solution was carefully matched in both the ligand and enzyme solutions. Mother liquor of phospholipids including DHPG (in water), DHPC (in DMSO), DHPE (in DMSO) (Avanti) with 200 mM concentration were prepared. Working buffer (20 mM DHPG, 50 mM DHPC, 10 mM DHPE) is prepared by diluting the mother liquor with buffer 100 mM Tris, 150 mM KCl, 10 mM CaCl_2_, pH 7.5.

### Molecular docking

Docking experiments were performed using Glide docking from Maestro 9.2013 (Schrödinger). Protein model from complex structure of hGIIE-compound 24 (PDB: 5WZU) was pre-processed using protein preparation wizard. Ligands (DHPE, DHPG, DHPC and DHPS) were prepared in Maestro and processed with ligand preparation wizard to add hydrogen atoms and generate perfect conformation with the OPLS_2005 force filed. Receptor grid generation was used to define docking space and to generate the grid box, and the grid box was generated around the compound 24. Docking experiment was performed with standard precision (SP) ligand docking. The docked model were ranked by GLIDE score.

### Inhibition analysis

Inhibition of wild type (WT) and mutated hGIIE was analyzed using sPLA_2_ assay kit as mentioned above. In the assay system, 5 µL assay buffer was replaced by 5 µL of the inhibitor. Different concentrations of compound 8, 14, 24, Me-Indoxam, and LY311727 were prepared. The final concentration of enzyme used per well are as following: WT: 6.42 µM; F5L: 6.4 µM; F97L: 10.48 µM; F5L_F97L: 6.73 µM; G6H: 10.91 µM; G6N: 7.56 µM; N21G: 32.86 µM; N21M: 22.14 µM; N21W: 30 µM. Control reactions without enzyme or inhibitor were carried out. Percent inhibition of every well were calculated. Curve was fitted to the log (inhibitor)-response variable slope and IC_50_ was calculated using GraphPad Prism 5 (GraphPad).

### Accession codes

Atomic coordinates and structure factors of the reported crystal structure have been deposited in Protein Data Bank (PDB) under the accession codes: 5WZM (apo-hGIIE_1); 5WZO (apo-hGIIE_2); 5WZS (hGIIE-compound 8); 5WZT (hGIIE-compound 14); 5WZU (hGIIE-compound 24); 5WZV (hGIIE-Me-Indoxam); 5WZW (hGIIE-LY311727).

## Electronic supplementary material

Supplementary information
