# Supplementary material for: Structural basis for functional selectivity and ligand recognition revealed by crystal structures of human secreted phospholipase A2 group IIE
Source: Sci Rep. 2017 Sep 7;7:10815. doi: 10.1038/s41598-017-11219-8 (PMC5589937; doi:10.1038/s41598-017-11219-8)
Supplement: Supplementary file 1 — Supplementary information [file 41598_2017_11219_MOESM1_ESM.pdf]

Structural basis for functional selectivity and ligand recognition revealed by crystal structures of human secreted phospholipase A_2 group IIE

Shulin Hou[1, 2], Tingting Xu[1, 3], Jinxin Xu[1], Linbing Qu[1], Yong Xu[4], Ling Chen[1] & Jinsong Liu[1, 4]*

[1] State Key Laboratory of Respiratory Disease, Guangzhou Institutes of Biomedicine and Health, Chinese Academy of Sciences, Guangzhou 510530, China.

[2] University of Chinese Academy of Sciences, Beijing 100000, China.

[3] School of Life Sciences, University of Science and Technology of China, Hefei 230026, China

[4] Guangdong Provincial Key Laboratory of Biocomputing, Institute of Chemical Biology, Guangzhou Institutes of Biomedicine and Health, Chinese Academy of Sciences, Guangzhou 510530, China.

* Correspondence:

Address: 190 Kaiyuan Avenue, Guangzhou Science Park, Guangzhou 510530, China.

Fax: +86 20 32015299.

E-mail addresses: liu_jinsong@gibh.ac.cn

SUPPLEMENTARY RESULTS

Supplementary Figure 1. **Multiple sequence alignment of hGIIE with other human sPLA$_2$s.** All sequences are from matured protein without signal pepetide, and without propeptide in the case of hGIB and hGX. Secondary structure elements of hGIIE are shown at the top. Residues are shown in white on the red background in case of strict identity. Residues with high similarity are colored in red and framed in blue. Multialignment was performed using the program multalign [1] and espript 3.0 [2].

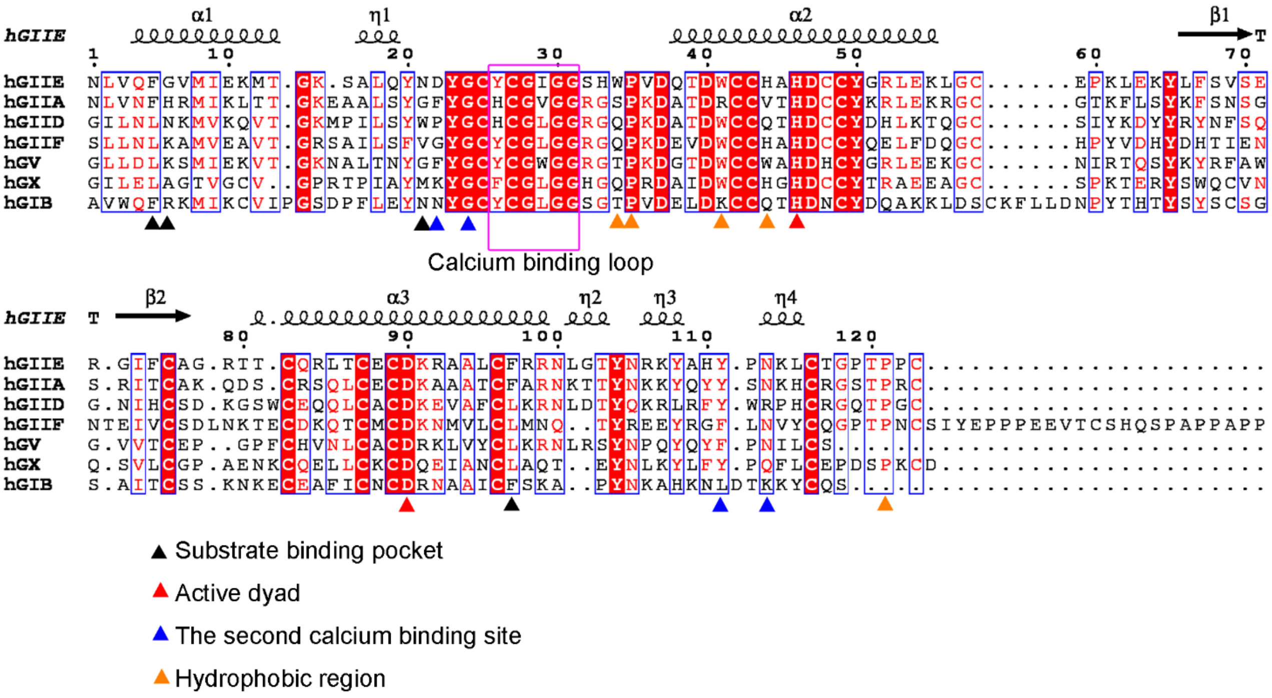

Supplementary Figure 2. **Comparison of hGIIE with other sPLA2s structures. (a)** Interfacial binding surface of hGIIE with compound 14, surface is colored by electrostatic potential (red to blue, -10 kT/e to 10 kT/e [3,4]). Compound 14 is shown as yellow stick. (**b**) Special hydrophobic core of hGIIE on the back of the interfacial binding surface. Residues in this region are labeled and shown in stick. (**c**) Interfacial binding surface of hGIIA with transition state analogue (hGIIA-TSA, pdb: 1POE). (**d**) Superposition of β-strand region of hGIIE, hGIIA (pink, pdb: 3U8B), hGIB (blue, pdb: 3ELO) and hGX (yellow, pdb: 1LE6). Residues between two β-strands of hGIIE are labeled and shown in stick. (**e**) The distance between α1 helix and the loop region between two β-strands for hGIIE(gray) and hGIIA (pink, pdb: 3U8B).

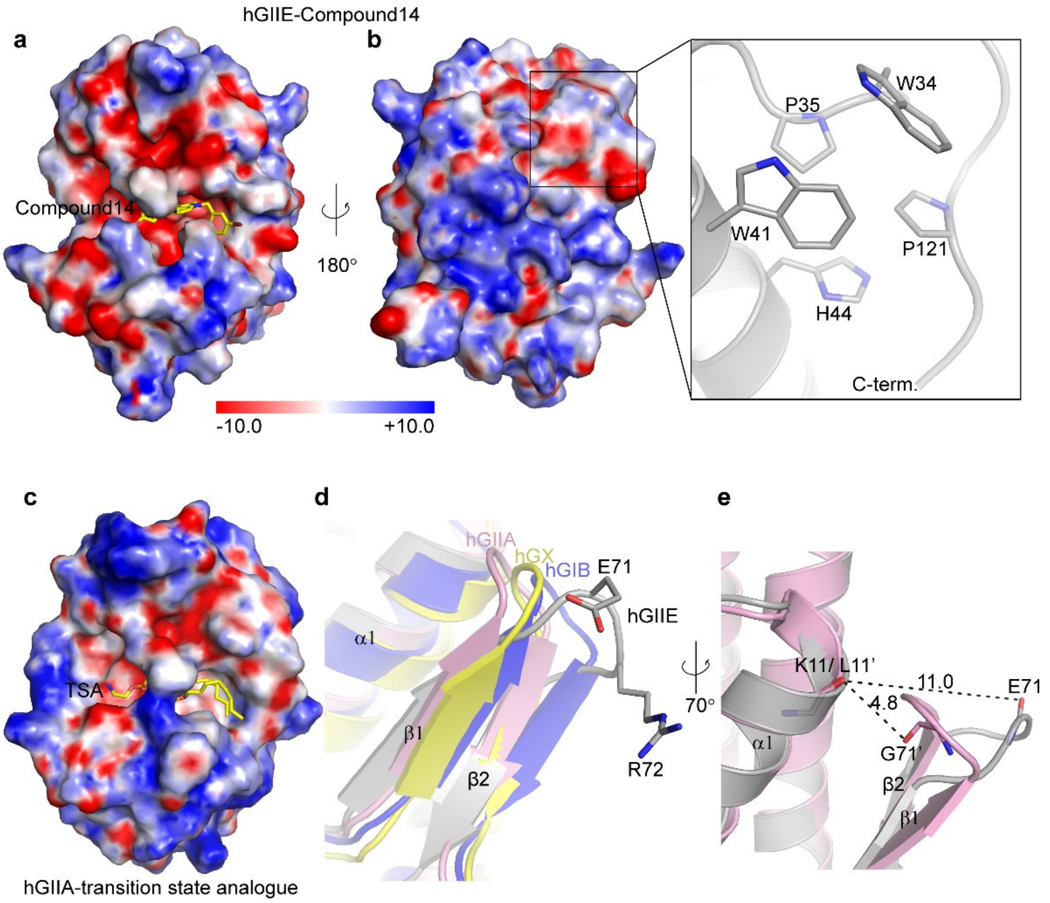

Supplementary Figure 3. **Superposition of the second calcium binding site of hGIIE (gray) and hGIB (blue, pdb: 3ELO) and hGX (yellow, pdb: 1LE6)**. Residues involved in the calcium binding sites are labeled and shown in line presentation. Water is shown in red sphere, calcium ions are shown in green spheres. hGIB and hGX have no second calcium binding site.

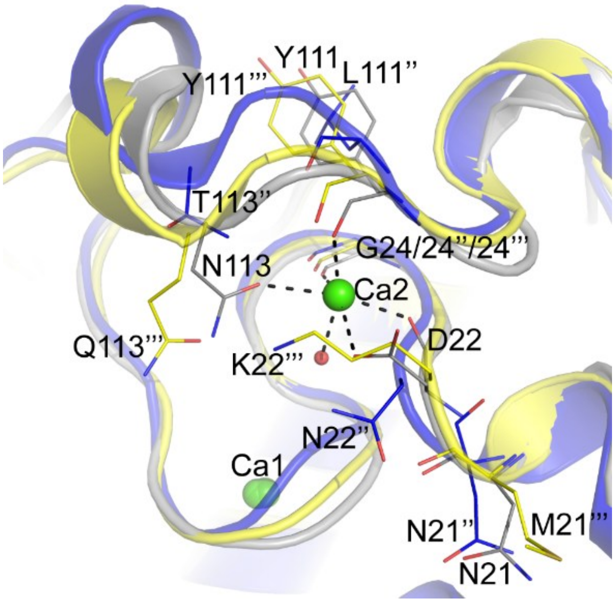

Supplementary Figure 4. **Enzymatic activity and kinetic parameters of wild type (WT) and mutant hGIIE.** Enzymatic activity of WT (3.80±0.12 U/mg) was defined as 100% relative activity. Data is shown as the means values ±s.d. (n=3) (Data details are shown in Supplementary Table. 2).

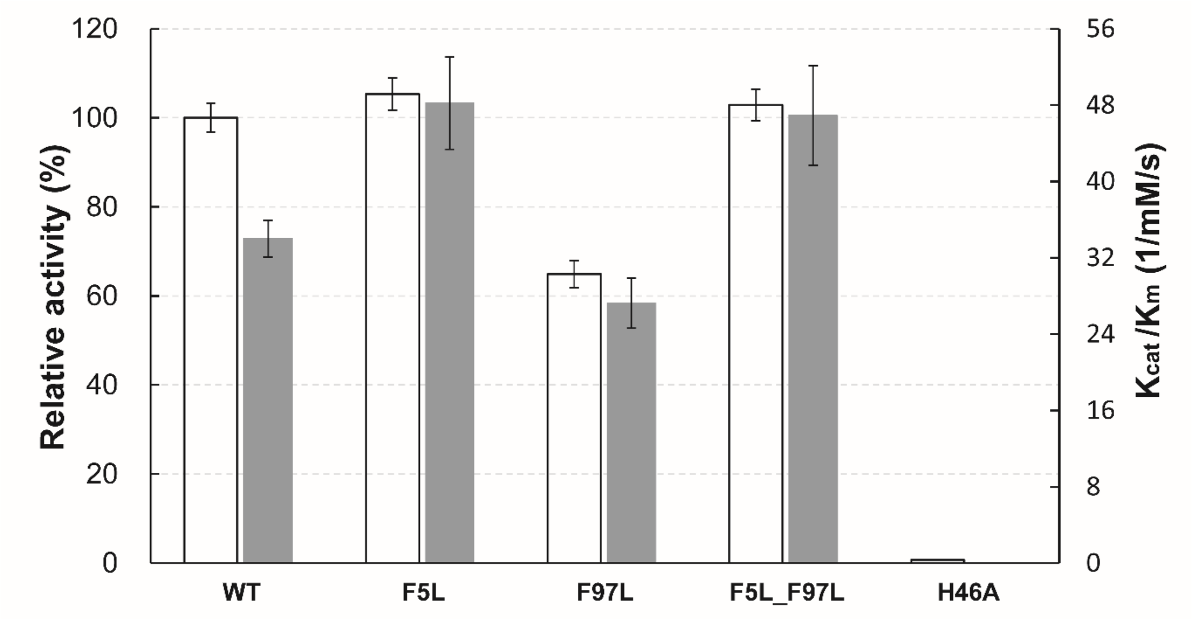

Supplementary Figure 5. **Inhibition data against WT and mutated hGIIE by LY311727, compound 8/14/24, and Me-Indoxam.** IC_{50} values for WT are labeled. Inhibition data against the mutant is shown as IC_{50} (mutant)/ IC_{50} (WT). Data is shown as the means values ±s.d. (n=2) (Data details are shown in Supplementary Table. 3).

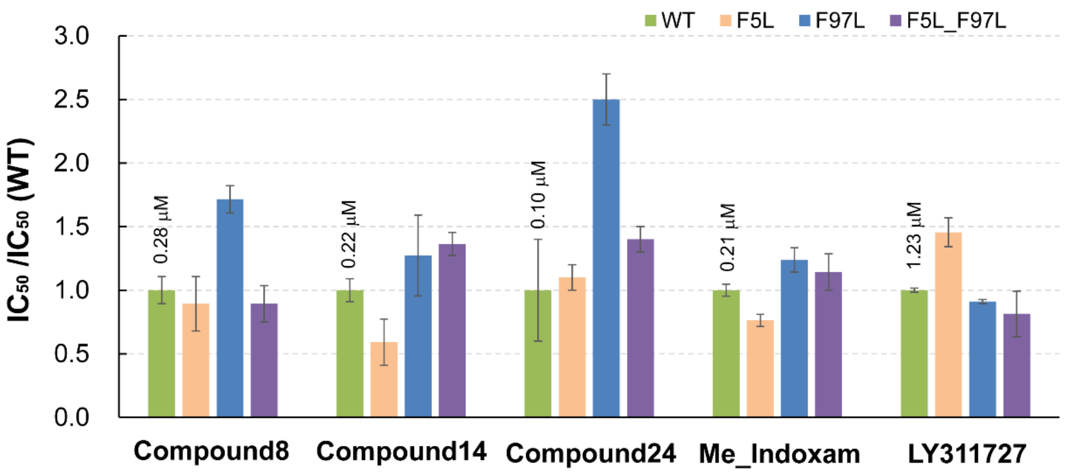

Supplementary Figure 6. **Docking model of hGIIE with 1, 2-dihexanoyl-sn-glycero-3-phosphor-L-serine (DHPS).** Hydrogen bond interaction is indicated by black dash lines, and residues involved are shown as stick.

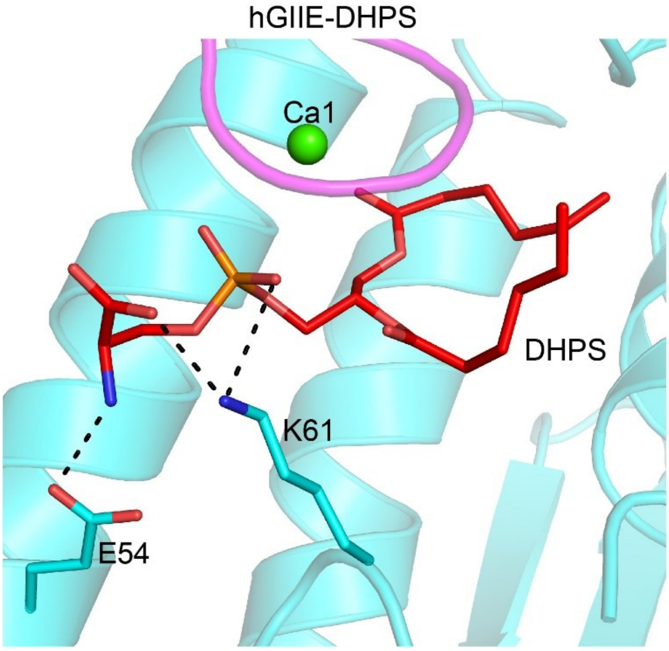

Supplementary Figure 7. **Schematic model of hGIIE with substrate and inhibitor.** **(a)** hGIIE and the tetrahedral intermediate; **(b)** hGIIE and compound 24. Atoms in protein are colored in blue, the water molecule participating in the hydrolyzation is shown in red, tetrahedral intermediate and compound 24 are colored in black. Interaction beteween protein and ligand is indicated by dash lines.

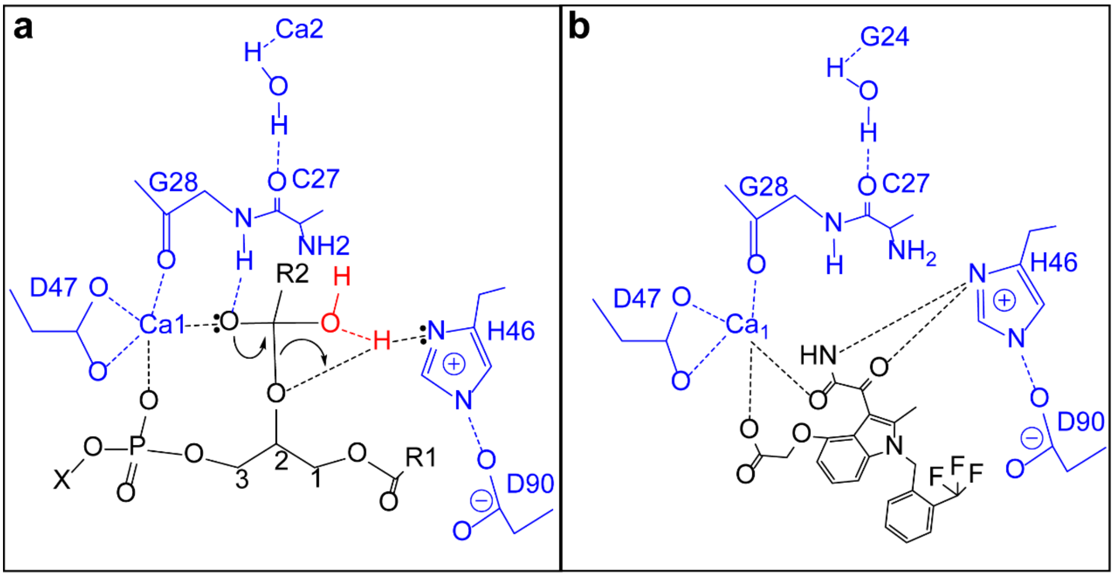

Supplementary Figure 8. **Structures of (a)** compound 8; **(b)** compound 14; **(c)** compound 24; **(d)** Me-Indoxam; **(e)** LY311727.

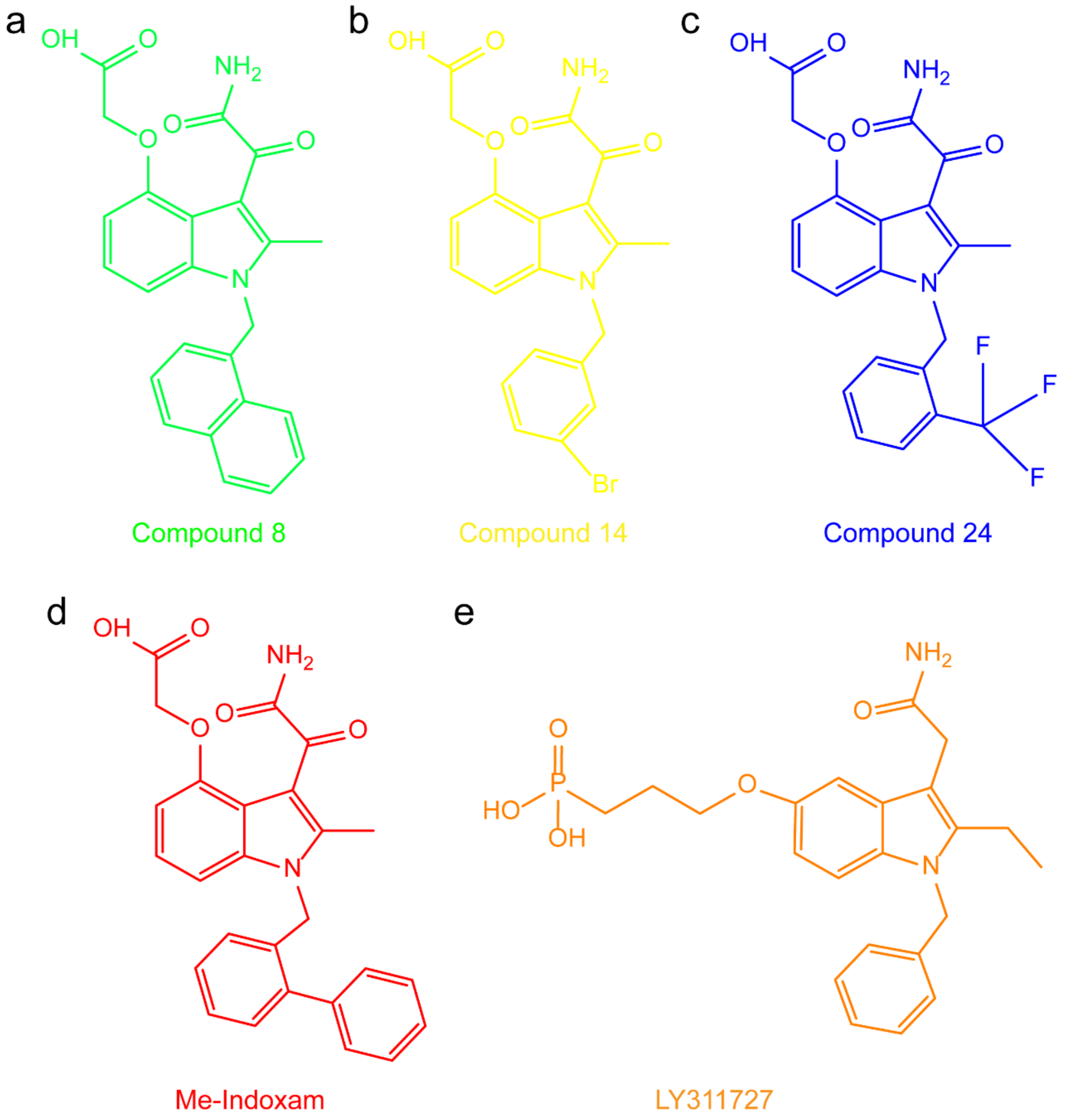

Supplementary Figure 9. **Structural comparison of compound 24 binding with WT hGIIE and N21G mutant hGIIE.** **(a)** Superposition of WT hGIIE with compound 24 (blue) and N21G mutant hGIIE with compound 24 (yellow, PDB:5Y5E); **(b)** Fo-Fc electron density map at 3 σ level around compound 24.

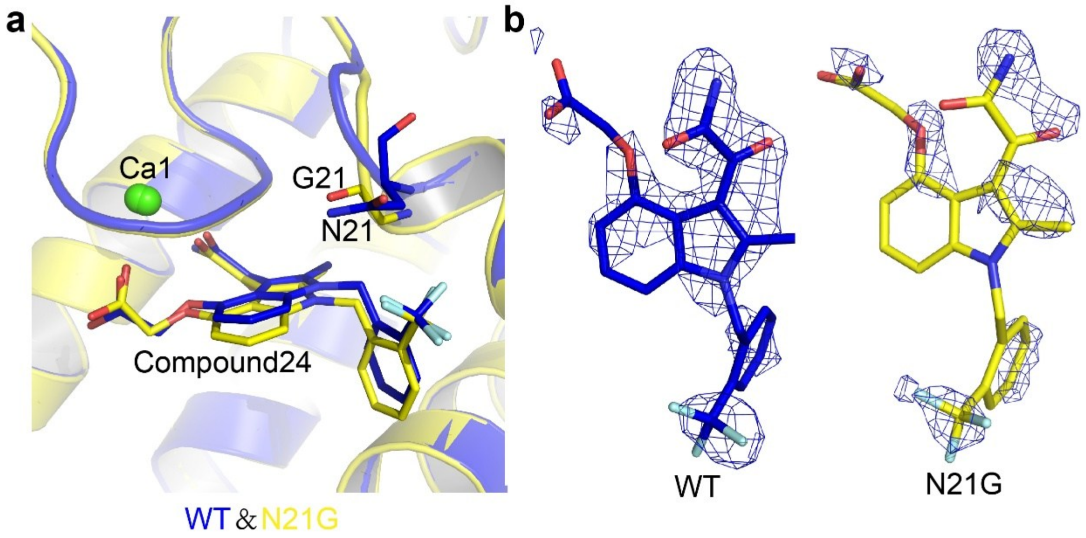

Supplementary Table 1. **Data collection and refinement statistics**

	apo_hGIIE_1<br>PDB: 5WZM	apo_hGIIE_2<br>PDB: 5WZO	hGIIE:Compound8<br>PDB: 5WZS	hGIIE:Compound14<br>PDB: 5WZT	hGIIE:Compound24<br>PDB: 5WZU	hGIIE: Me-indoxam<br>PDB: 5WZV	hGIIE: LY311727<br>PDB: 5WZW
**Data collection**							
Space group	$P2_122$	$P2_122$	$P2_122$	$P2_122$	$P2_122$	$P2_122$	$P2_122$
Cell dimensions							
a, *b*, *c* (Å)	48.51, 60.46, 63.59	49.06, 60.91, 63.20	49.01, 61, 63.30	48.93, 60.68, 63.38	48.63, 60.47, 63.50	49.08, 61.05, 63.33	48.85, 61.22, 63.33
α=β=γ (°)	90	90	90	90	90	90	90
Resolution (Å)	43.82-2.00 (2.06-2.00)	18.76-1.90 (1.94-1.90)	18.47-2.30 (2.39-2.30)	18.47-2.40 (2.49-2.40)	18.48-2.20 (2.27-2.20)	19.37-2.20 (2.27-2.20)	17.38-1.95 (2.00-1.95)
R_{merge}	0.105 (0.561)	0.100 (0.664)	0.117 (0.557)	0.115 (0.573)	0.091 (0.386)	0.119 (0.620)	0.046 (0.190)
$I / \sigma I$	11.7 (3.7)	16.6 (2.7)	17.0 (4.4)	16.8 (4.5)	20.2 (5.7)	13.9 (3.4)	19.6 (4.7)
Completeness (%)	100 (100)	98.5 (85.3)	99.8 (100)	99.8 (100)	99.8 (100)	99.2 (99.9)	99.8 (100)
Redundancy	7.1 (7.3)	8.8 (4.5)	8.9 (9.7)	9.2 (10.2)	9.9 (9.4)	7.1 (7.6)	4.5 (3.3)
**Refinement**							
Resolution (Å)	43.82-2.00	18.76-1.90	18.47-2.30	18.47-2.40	18.48-2.20	19.37-2.20	17.38-1.95
No. reflections	12500	14477	8433	7405	9489	9553	13626

R_{work} / R_{free}	18.46/22.60	20.82/24.47	21.50/26.55	21.86/26.74	19.54/23.66	20.82/24.04	20.41/25.52
No. atoms							
Protein	963	996	985	979	993	985	985
Ligand/ion	26	42	43	39	44	52	50
Water	105	194	66	50	91	102	163
B-factors							
Protein	27.77	17.14	30.04	32.16	28.07	29.14	18.02
Ligand/ion	40.09	20.76	52.59	49.96	56.67	55.01	45.82
Water	35.88	26.01	26.95	27.44	27.94	30.48	25.68
R.m.s. deviations							
Bond lengths (Å)	0.0158	0.0152	0.0093	0.0105	0.0141	0.0115	0.0089
Bond angles (°)	1.7486	1.7711	1.3598	1.4207	1.5809	1.5230	1.4460
Ramachandran plot (%)							
Most favored	97.48	97.52	98.35	99.17	98.35	97.52	99.17
Disallowed	0	0	0	0	0	0	0

One crystal was used for data collection. Values in parentheses are for highest-resolution shell.

Supplementary Table 2. **Enzymatic activity and kinetic parameters of wild type and mutant hGIIE.** Data is shown as the means values ±s.d. (n=3).

Region	hGIIE	Activity (U/mg)	Relative Activity (%)	K_m (mM)	K_{cat} (1/s)	K_{cat}/ K_m (1/mM/s)
	WT	3.80±0.12	100.00±3.23	0.90±0.10	30.60±1.76	34.01±1.93
Substrate binding pocket	F5L	4.00±0.14	105.34±3.61	0.77±0.15	37.19±3.46	48.22±4.85
	F97L	2.47±0.12	64.91±3.07	0.76±0.14	20.62±1.82	27.25±2.63
	F5L_F97L	3.91±0.13	102.91±3.54	0.63±0.13	29.70±2.67	46.91±5.22
	G6H	3.14±0.05	82.59±1.42	1.22±0.21	33.77±3.29	27.75±2.09
	G6N	4.14±0.13	108.93±3.50	0.90±0.15	36.24±3.03	40.43±3.28
	N21G	0.47±0.02	12.49±0.61	0.88±0.16	8.13±0.75	9.25±0.84
	N21M	0.85±0.04	22.49±1.03	0.77±0.15	9.32±0.86	12.05±1.20
	N21W	0.57±0.04	15.01±0.97	1.39±0.32	11.60±1.60	8.37±0.80
	H46A	0.03±0.00	0.69±0.01	-	-	-
The first calcium binding site	D47A	0.01±0.00	0.19±0.03	-	-	-
The second calcium binding site	D22A	0.53±0.05	14.02±1.35	0.45±0.04	3.83±0.13	8.52±0.43
	D22K	1.07±0.10	28.14±2.74	0.60±0.05	8.68±0.32	14.43±0.67
	N113A	1.54±0.04	40.50±1.16	1.51±0.41	16.22±2.65	10.78±1.15
	N113K	0.91±0.06	23.98±1.48	0.62±0.04	7.11±0.20	11.42±0.41

Supplementary Table 3. **Inhibition data against wild type and mutant GIIE for LY311727, compound 8/ 14 / 24 and Me-indoxam.** Data is shown as the means values ±s.d. (n=2).

hGIIE	IC_{50} (μM)				
	Compd8	Compd14	Compd24	Me-indoxam	LY311727
WT	0.28±0.03	0.22±0.02	0.10±0.04	0.21±0.01	1.23±0.02
F5L	0.25±0.06	0.13±0.04	0.11±0.01	0.16±0.01	1.79±0.14
F97L	0.48±0.03	0.28±0.07	0.25±0.02	0.26±0.02	1.12±0.02
F5L_F97L	0.25±0.04	0.30±0.02	0.14±0.01	0.24±0.03	1.00±0.22
G6H	3.76±0.86	0.88±0.31	0.54±0.03	0.30±0.02	14.31±0.39
G6N	0.63±0.05	0.23±0.03	0.27±0.03	0.14±0.02	3.07±0.42
N21G	0.63±0.02	1.73±0.05	2.39±0.40	1.84±0.03	5.13±1.07
N21M	1.05±0.02	0.84±0.03	0.83±0.22	0.88±0.01	5.98±0.40
N21W	1.27±0.22	1.94±0.22	1.87±0.18	1.44±0.13	17.97±1.37

Supplementary Table 4. **Primers for hGIIE gene cloning and mutation**

Sequence	Forward primer	Reverse primer
hGIIE	CCGGAATTCAATCTGGTTCAGTTTGGCG	CTAGTCTAGATCAGCACGGCGGCGTCGGA
F5L	CTGGTTCAG**TTG**GGCGTTATGATC	GATCATAACGCC**CAA**CTGAACCAG
G6H	GTTCAGTTT**CAC**GTTATGATCGAA	TTCGATCATAAC**GTG**AAACTGAAC
G6N	GTTCAGTTT**AAC**GTTATGATCGAA	TTCGATCATAAC**GTT**AAACTGAAC
N21G	CTGCAGTAT**GGT**GATTACGGCTGC	GCAGCCGTAATC**ACC**ATACTGCAG
N21M	CTGCAGTAT**ATG**GATTACGGCTGC	GCAGCCGTAATC**CAT**ATACTGCAG
N21W	CTGCAGTAT**TGG**GATTACGGCTGC	GCAGCCGTAATC**CCA**ATACTGCAG
D22A	CTGCAGTATAAC**GCT**TACGGCTGCT	AGCAGCCGTA**AGC**GTTATACTGCAG
D22K	ACTGCAGTATAAC**AAG**TACGGCTGC	GCAGCCGTA**CTT**GTTATACTGCAGT
H46A	TGTCATGCT**GCT**GACTGCTGTTAC	GTAACAGCAGTC**AGC**AGCATGACA
D47A	GTCATGCTCAC**GCT**TGCTGTTACG	CGTAACAGCA**AGC**GTGAGCATGAC
F97L	GCCCTGTGT**TTG**CGTCGCAATCTG	CAGATTGCGACG**CAA**ACACAGGGC
N113A	CTCACTATCCG**GCT**AAACTGTGTA	TACACAGTTT**AGC**CGGATAGTGAG
N113K	TCCG**AAG**AAACTGTGTACGGGTCCGAC<br>GCCGCCGTGCTGATCTAGACTAG	CTAGTCTAGATCAGCACGGCGGCGTCGGA<br>CCCGTACACAGTTTC**TTC**GGA

1 Corpet, F. Multiple sequence alignment with hierarchical clustering. *Nucleic acids research* **16**, 10881-10890 (1988).

2 Robert, X. & Gouet, P. Deciphering key features in protein structures with the new ENDscript server. *Nucleic acids research* **42**, W320-324, doi:10.1093/nar/gku316 (2014).

3 Baker, N. A., Sept, D., Joseph, S., Holst, M. J. & McCammon, J. A. Electrostatics of nanosystems: application to microtubules and the ribosome. *Proceedings of the National Academy of Sciences of the United States of America* **98**, 10037-10041, doi:10.1073/pnas.181342398 (2001).

4 Dolinsky, T. J., Nielsen, J. E., McCammon, J. A. & Baker, N. A. PDB2PQR: an automated pipeline for the setup of Poisson-Boltzmann electrostatics calculations. *Nucleic acids research* **32**, W665-667, doi:10.1093/nar/gkh381 (2004).
